# Juvenile zebrafish (*Danio rerio*) are able to recover from lordosis

**DOI:** 10.1038/s41598-022-26112-2

**Published:** 2022-12-13

**Authors:** A. Printzi, D. Mazurais, P. E. Witten, L. Madec, A.-A. Gonzalez, X. Mialhe, J.-L. Zambonino-Infante, G. Koumoundouros

**Affiliations:** 1 Biology Department, University of Crete, Crete, Greece; 2 IFREMER, University of Brest, CNRS, IRD, LEMAR, 29280 Plouzané, France; 3 Department of Biology, Gent University, Gent, Belgium; 4 MGX-Montpellier GenomiX, CNRS, INSERM, Univ. Montpellier, Montpellier, France

**Keywords:** Molecular biology, Zoology

## Abstract

Haemal lordosis, a frequent skeletal deformity in teleost fish, has long been correlated with increased mechanical loads induced by swimming activity. In the present study, we examine whether juvenile zebrafish can recover from haemal lordosis and explore the musculoskeletal mechanisms involved. Juveniles were subjected to a swimming challenge test (SCT) that induced severe haemal lordosis in 49% of the animals and then immediately transferred them to 0.0 total body lengths (TL) per second of water velocity for a week. The recovery from lordosis was examined by means of whole mount staining, histology and gene expression analysis. Results demonstrate that 80% of the lordotic zebrafish are capable of internal and external recovery within a week after the SCT. Recovered individuals presented normal shape of the vertebral centra, maintaining though distorted internal tissue organization. Through the transcriptomic analysis of the affected haemal regions, several processes related to chromosome organization, DNA replication, circadian clock and transcription regulation were enriched within genes significantly regulated behind this musculoskeletal recovery procedure. Genes especially involved in adipogenesis, bone remodeling and muscular regeneration were regulated. A remodeling tissue-repair hypothesis behind haemal lordosis recovery is raised. Limitations and future possibilities for zebrafish as a model organism to clarify mechanically driven musculoskeletal changes are discussed.

## Introduction

Skeletal deformities are acknowledged as a valuable welfare index for reared and laboratory teleost fish, often correlated with the quality of the rearing conditions^1,2^. They can negatively affect the biological performance and behavior of the animals^3^. Either as developmental deviations from the normal pattern (embryonic and larval periods^4,5^) or post-metamorphic deformations of initially normal bones (e.g., swimming induced lordosis^6^), deformities are mostly considered as irreversible morphological defects. Recently though, an increasing number of observations report a growth-correlated recovery potential of certain types. The recovery of gill cover abnormalities, through regeneration or repair, is reported to be dependent on their intensity^7,8^. In Atlantic salmon, not only are fused vertebral centra able to remodel in one normal-shaped body^9^ but also hyper-dense and compressed vertebrae can recover^10^. Remarkably, around 44–74% and 60% of lordotic seabream and seabass juveniles respectively, achieved a partial to complete reshaping of the abnormal lordotic vertebral elements during the on-growing period^11,12^.

Bone is capable of dynamic changes over time, under the orchestrated function of formation and resorption^13^. Responding to external stimuli, osteoblasts, osteoclasts and osteocytes are capable of retaining bone integrity through time and injuries^14^. This dynamic procedure of bone remodeling is known to be correlated with mechanical loads^15^. The ability of the skeleton to respond and adapt to mechanical stimuli and a high degree of phenotypic plasticity, is shared among species with cellular (most vertebrate species) and acellular (advanced teleosts) bone^16^. Even during early ontogenetic stages, increased mechanical loads derived from intense swimming exercise can trigger skeletogenesis in zebrafish^17^. In adult zebrafish, osteoblast number, bone volume and vertebrae mineralization increase in response to swimming exercise^18^. Furthermore, the ability of vertebral bodies to surpass from normal to a severe lordotic phenotype within a few days of intense swimming exercise^1^, highlights zebrafish early juveniles as suitable model to study bone’s transition process between normal and abnormal.

Although haemal lordosis shares common anatomical features between different teleost species (*red sea bream*^19^, *sea bass*^20^, *zebrafish*^1^), the nature of the musculoskeletal responses during these short transitions between normal–lordotic–normal, remains unclear. Hypotheses already stated, focus on the view that lordosis induction isan adaptive modeling of the vertebral column to adjust to the compressive forces created by alternated lateral bending^21^. Altered pattern of vertebral loads derived from altered strains^22^ accompanied by axial muscle adaptations^23^ could thus possibly explain the fast changes. On the cellular level, taking into consideration the direct relationship of bone and muscle in the haemal region affected, the possibility of a more complicated response exists. The scenario of skeletal muscle and/or bone injury and repair would include an initial immune inflammatory response, attraction of pro-inflammatory factors, removal of the damaged tissue, recruitment of stem cells to the cite, differentiation according to the repair needs^24^. Several molecules such as pro-inflammatory cytokines and growth and angiogenic factors have been reported to interfere with bone healing^25,26^. Simultaneously, genes regulating metabolism and biogenesis of mitochondria, oxidative stress and increasing cholesterol and lipid synthesis^27^ have been described to participate in muscle injury response.

The aims of the present study were (a) to address whether juvenile zebrafish can recover from haemal lordosis through histological approaches, and (b) to explore possible changes at the transciptomic level of this fast musculoskeletal remodeling from lordotic to a normal phenotype.

## Results

### Lordosis incidents recover within a week

**Figure 1:**
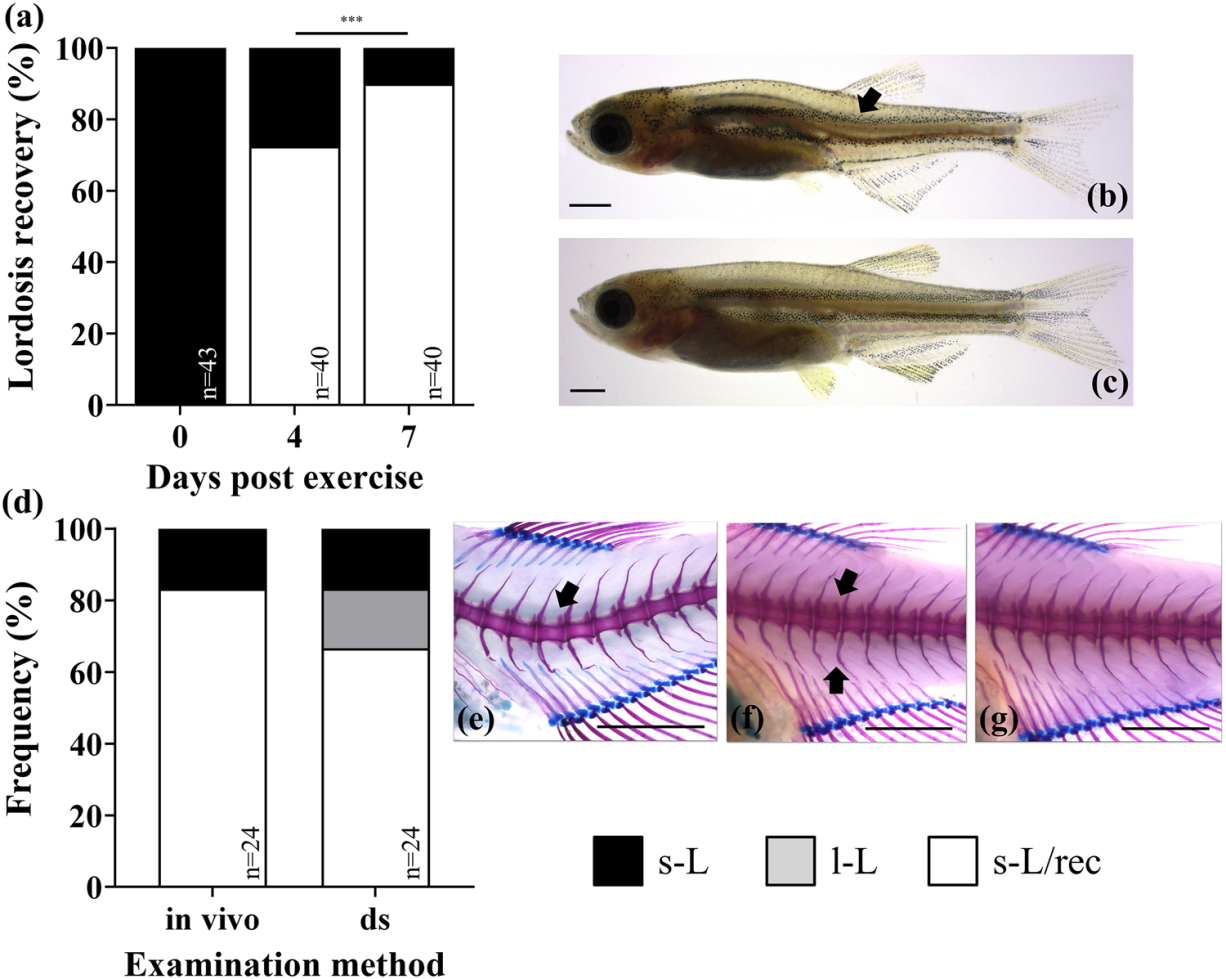
Recovery frequency of the external morphology of the severe lordotic individuals at 4 and 7 dpe (**a**–**c**) and valorization of the scoring methodology by means of double staining (**d**–**g**) at the end of the recovery period (7 dpe). (**b**, **c**) Representative case of the transition of a juvenile from a typical lordotic (s-L) to a recovered (s-L/rec) external morphology respectively. (**e**–**g**) Representative cases of the internal bone structure of a severe lordotic (s-L), a light lordotic (l-L) and a recovered (s-L/rec) individual after double staining (ds) respectively. Scale bars equal to 1 mm. n, individual number assessed. Asterisks indicate significant differences (*p* < 0.05).

Juvenile zebrafish were forced to swim continuously for 4-days at 56% of their critical swimming speed. At the end of this period, 49.4% of fish had severe lordosis (s-L), 35.6% had light lordosis (l-L), and 14.9% had no signs of lordosis (N) based on the external phenotype. Subsequently, all phenotypes were allowed to recover from the swimming exercise for 7 days under 0.0 TL s^−1^ water velocity (TL, Total Length). A high recovery rate from haemal lordosis was observed in zebrafish juveniles (Fig. 1a). The percentage of the externally recovered individuals from severe lordosis (s-L/rec) was significantly increased from 73 to 90% within only 4 and 7 days, respectively, of swimming under almost 0.0 TL s^−1^ water velocity (Fig. 1a, *p* < 0.05). Recovered individuals presented a completely normal external morphology, lacking the typical lordotic bending of the vertebral column (Fig. 1b, c). These results were validated by the double staining of 4 s-L and 20 s-L/rec juveniles at the end of the trials (7 dpe, days post exercise) (Fig. 1d–g). After staining, all s-L juveniles presented a bent vertebral column (Fig. 1e), whereas 4 out of 20 fish (20%) with a normal external morphology presented light internal lordosis characteristics (slight bending of some vertebral bodies in the haemal region along with inverted spine orientation, Fig. 1f), instead of a recovered internal structure (Fig. 1g).

### Complete bone recovery is achieved with internal distortion

**Figure 2:**
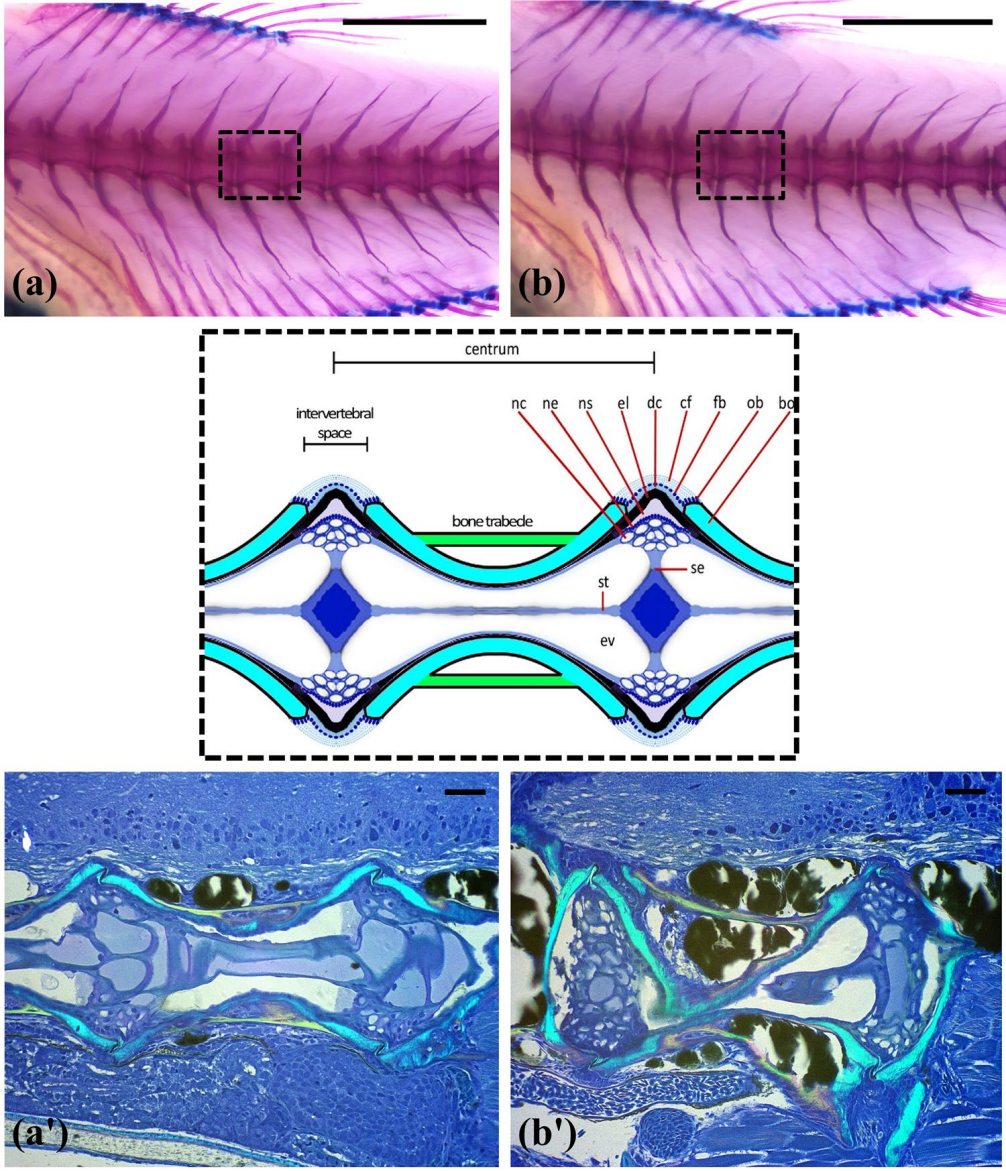
Comparison of a normal (N) (**a**, **a**′) and a recovered fish (s-L/rec) (**b**, **b**′) 4 days post exercise. Stained pictures (**a**, **b**) and parasagittal sections (**a**′, **b**′) of the vertebral bodies located in the center of haemal lordosis. Bo, bone of the vertebral body endplate. Cf, collagen type I fiber bundles. Dc, dense collagen type I matrix. El, elastin. Ev, extracellular vacuole. Fb, fibroblasts. Nc, vacuolated notochord cells. Ne, notochord epithelium. Ns, notochord sheath. Ob, osteoblasts. Se, septum. St, notochord stan. Scale bars equal to 1 mm (**a**, **b**) and 50 μm (**a**′, **b**′).

A normal (N) vertebral column did not show differences compared to a recovered one (s-L/rec), concerning vertebra body shape and orientation at four dpe (Fig. 2a, b). The images presented are representative examples of the three individuals examined from each group. A recovered vertebral centrum revealed, however, distorted internal bone structures but no fractures (Fig. 2b′). Despite the internal distortions, the external shape of the vertebral centrum was preserved. The notochord and its sheath in the intervertebral spaces remained intact. Around the bone and inside the bone marrow spaces adipose tissue was abundant. The orientation of collagen fibers in the bone of the vertebral centra and in the connecting spongiosa was different between the normal (N, Fig. 2a′) and the recovered (s-L/rec, Fig. 2b′) individuals.

### Recovered haemal region is under active tissue remodeling

RNA sequencing, at 4 days post SCT, highlighted 1982 (*p* value < 0.05) and 973 genes (*p* value < 0.01) within the total 23,168, differentially expressed between normal (N) and recovered (s-L/rec) individuals (supplementary data 1). Out of the 973 genes, 562 were up-regulated and 411 were down-regulated in the s-L/rec group. The enriched biological processes GO associated to the 973 genes mainly relied on chromosome organization, response to stress, circadian regulation of gene expression, response to hypoxia, DNA metabolic process, and regulation of transcription (Table 1, supplementary data 2). Within the regulated genes involved in chromosome organization (n = 30), 5 MCM family genes (*mcm 2,3,5,6,9*) playing a central role in the initiation of DNA replication during the G1/S phase were found. Included in “response to stress” GO and among other several *hsp* genes, we found some genes involved in the response to hypoxia (e.g. *egln2*, *hif* genes) and to photoperiodic signal (e.g. *ccry-dash, per* genes), in the regulation of cell proliferation (e.g. *rad* genes, *dusp1*) and in inflammation (e.g. *cxcl* genes). “DNA metabolic process” GO included among others *mcm* genes mentioned above, polymerase genes (e.g. *pold1, pold2*) and factors involved in DNA repair (e.g. *mer11a*, *ddb2*). “Regulation of transcription, DNA-templated” GO included mainly transcription factors involved in the activation and/or repression of gene expression (e.g. *med17, nfyal, runx1t1, mitfa, fosab, nfatc1, hox* and *sox* genes*)*.

**Table 1:** Enriched GO (biological processes, cell component, molecular pathways; FDR < 0.05) associated with genes differentially expressed (*p* < 0.01) in the haemal region between the normal (N) and recovered (s-L/rec) groups.

GO biological processes	nGenes	EnrichFDR	Fold enrich	Genes (off. gene symbols)
**Biological process**				
Chromosome organization	30	0.02	2.13	*kdm5bb wrap73 hcfc1b hira mcm9 mcm5 mcm3 chd4b top2b ing3 rtel1 histh1l hmgn3 h1-0 rad50 nasp h1-10 l3mbtl2 chaf1b mcm6 hcfc2 prkdc top1 hdac4 brd3b mcm2 uhrf1 anxa1c msh6 mre11a*
Response to stress	67	0.04	1.56	*cry-dash ube2al hsp90b1 rpa1 acsl4a sel1l hif1aa csrp1a vim thbs1b ptger2a hsc70 prkag2a atf6 cirbpb ercc4 keap1a sbno2 pdia4 rad17 eya2 cry5 herpud2 stat3 prnpa hmox1a pold1 hsp70.1 dnaja1 fosab per2 rtel1 cxcl12a rad50 xpc hspb1 ddb2 foxo3b pparda lepb crip1 uggt1 hsp70l per1a mcm6 sik1 eif2ak3 sik2b egln2 ercc8 arhgef10lb cxcl18b prkdc spring1 nfe2l2b pold2 hmgb1a dusp1 hif1al f8 cxcl8b.1 mcm2 uhrf1 cav1 anxa1c msh6 mre11a*
Circadian reg. of gene expression	7	0.04	5.59	*per3 per1b cry5 per2 per1a ciarta cry2*
Response to hypoxia	8	0.04	4.38	*hsp90b1 hif1aa atf6 hmox1a foxo3b eif2ak3 egln2 hif1al*
DNA metabolic process	30	0.04	1.95	*cry-dash ube2al rpa1 ercc4 dnase1l4.1 rad17 eya2 cry5 mcm5 mcm3 pold1 dnmt1 top2b rtel1 h1-0 rad50 xpc ddb2 cebpb dffa mcm6 ercc8 prkdc orc6 top1 pold2 mcm2 uhrf1 msh6 mre11a*
Regulation of transcription, DNA-templated	109	0.04	1.37	*nfyal phc1 runx1t1 mitfa bach2b hif1aa med17 rb1 mycn zmynd8 rbl1 gata2b smad3b per3 sox19a prkcbp1l dpf2 per1b hcfc1b atf6 foxn3 hira ikzf1 znf710a bicral sbno2 taf5 igf2a cnot3b jdp2b safb foxd3 stat3 znf395b max ssbp3b klf11a fosab prrx1a zgc:65,895 cpsf1 per2 nfatc1 hoxa13b hmgn3 rybpa cbx7a ccar2 tefa pdcd4b cebpb foxo3b cux1b sox9b egr2a etv5b pparda ahrrb hsf2 vgll2b etv2 l3mbtl2 ssbp4 si:dkey-27c15.3 per1a snai1a hcfc2 nr1d4b znf740b rreb1a ftr80 si:ch211-132b12.7 ifrd1 si:ch211-261n11.5 foxi2 arid6 bnc2 hoxc12a hmga1b si:dkey-20i20.8 mical2a irf3 zgc:174,310 rnf6 nkx3-1 supt20 mgaa si:ch211-160f23.5 fosl1b nfil3-6 tshz3a alx4a deaf1 nfe2l2b tefb zmiz2 sox4b hmgb1a foxo1a hif1al ppargc1b cry2 jazf1a sirt6 si:ch73-21k16.1 uhrf1 hivep1 e2f1 jak2a*

Specifically, the biological process ontologies related to circadian rhythm, striated muscle cell development and response to abiotic stimulus were enriched within genes up-expressed in the recovered fish (Table 2, supplementary data 3). Among the regulated genes involved in circadian rhythm are included several members of the period gene family (per3, per1b, per2, per1a), cry5, ciarta and mitfa. The regulated genes listed in “response to abiotic stimulus” GO included several genes involved in entrainment of circadian clock by photoperiod and factors involved in the response to hypoxia (hsp90b1, hif1aa, atf6, hmox1a, foxo3b, eif2ak3, egln2, hif1al). The “striated muscle cell development” ontological group included especially genes involved in myofibril assembly (ttn.1, csrp1a, klhl41b, cxcl12a, tnnt1, ppp2r3a, smyd1b, asb2b). The over-representation of factors expressed in muscular cells within the genes up-expressed in the S-group was confirmed by numerous PDZ (e.g. ldb3b), LIM (e.g. csrp1a) and PDZ and LIM domain-encoding genes (e.g. pdlim3b,4,5b) as well as other members of the actin filament binding protein family present in the enriched cell component GO (Table 2, supplementary data 2).

**Table 2:** Enriched GO (biological processes, FDR < 0.01) associated with genes significantly up- expressed in the heamal region in the recovered individuals (s-L/rec).

Pathway	nGenes	Enrich. FDR	Fold enrich	Genes (off. gene symbols)
**Biological process**				
Circadian rhythm	9	0.01	5.36	*mitfa per3 per1b cry5 per2 per1a ciarta si:ch211-132b12.7 cry2*
Striated muscle cell development	8	0.03	4.95	*ttn.1 csrp1a klhl41b cxcl12a tnnt1 ppp2r3a smyd1b asb2b*
Response to abiotic stimulus	17	0.01	3.05	*hif1aa per3 per1b cirbpb cry5 hmox1a per2 tefa hspb1 foxo3b crip1 per1a egln2 piezo1 hif1al cry2 trpm1b*
**Cell component**				
Sarcoplasmic reticulum	6	< 0.01	11.69	*casq2 itpr2 jph2 atp2a2a jph1b si:ch211-266g18.10*
Actomyosin	5	< 0.01	8.66	*pdlim3b daam1a pdlim5b pdlim4 ldb3b*
Z disc	9	< 0.01	6.68	*csrp1a casq2 pdlim3b pdlim5b myoz2b myoz3a pdlim4 fhl1a ldb3b*
I band	9	< 0.01	6.52	*csrp1a casq2 pdlim3b pdlim5b myoz2b myoz3a pdlim4 fhl1a ldb3b*
Myofibril	15	< 0.01	5.84	*tnni2a.2 csrp1a casq2 pdlim3b pdlim5b myoz2b tnnt1 tnni1b si:ch211-125o16.4 myoz3a pdlim4 fhl1a smyd1b ldb3b tpm2*
Actin filament	7	< 0.01	5.59	*pdlim3b pdlim5b gas2l3 pdlim4 tpm1 ldb3b tpm2*
Intermediate filament	6	< 0.01	4.25	*vim neflb lmna krt94 nefla krt8*
Supramolecular fiber	25	< 0.01	2.73	*tnni2a.2 csrp1a casq2 vim ndel1a neflb lmna pdlim3b pdlim5b myoz2b tnnt1 krt94 gas2l3 tnni1b si:ch211-125o16.4 nefla krt8 myoz3a pdlim4 fhl1a tpm1 smyd1b ldb3b zgc:153,426 tpm2*
Actin cytoskeleton	18	< 0.01	2.58	*tnni2a.2 cfl2 pdlim3b daam1a pdlim5b myoz2b tnnt1 gas2l3 tnni1b myo1cb myoz3a pdlim4 myo19 tpm1 ldb3b antxr2a tpm2 smyhc2*
Mitochondrion	31	< 0.01	1.91	*got2b prelid3b clu ndufv2 oxct1a pdk2a ndufaf1 slc25a4 bnip3lb ndufs1 flvcr1 ogdha ndufa7 foxo3b vdac1 pdhx pptc7b pdk4 cpt1b ino80db clpxb bbc3 si:dkeyp-72g9.4 gpam letm2 prodha fars2 cs si:ch73-21k16.1 apooa*

The enriched biological process GO of the down-expressed genes in the recovered fish relied especially on chromosome organization and DNA conformation change (Table 3, supplementary data 3). Among the 30 genes involved in chromosome organization that were found to be regulated between the two groups, 25 were down-expressed in the s-L/rec group. These genes included among others MCM member family genes that play key roles in DNA replication (*mcm2,3,5,6* and *orc6* genes involved the G1 to S phase transition), factors involved in the DNA metabolic process including DNA polymerase (e.g. *pold1, pold2*), members of the MRE11 complex (*rad50*, *mre11*) and genes involved in histone assembly (e.g. *chafb1, l3mbtl2, nasp*). Among the enriched biological GO genes were also found implicated in the regulation of DNA-templated transcription. Most of these are transcription factors involved in the regulation of different biological processes including the development, proliferation and/or differentiation of various cell types (e.g. *rb1, rb1l, gata2b, uhrf1, hmgb1, snai1a, foxd3, sox4b, hoxa13b, fosab*). Our analysis of enriched cell component GO within the down-expressed genes in the S-group revealed also over representation of genes involved in DNA repair complex (*ercc4, ercc8, msh6*). Over representation of actors involved in both the biological process related to the cellular response to stress and associated to endoplasmic reticulum (e.g. *hsp90b1, sel1l, atf6, eif2ak3*) was also observed within genes down expressed in the recovered group.

**Table 3:** Enriched GO (biological processes, FDR < 0.05) associated with genes significantly down-expressed in the heamal region in the recovery (s-L/rec) group.

Pathway	nGenes	Enrich. FDR	Fold enrich	Genes (off. gene symbols)
**Biological process**				
DNA replication initiation	5	< 0.01	15	*mcm5 mcm3 mcm6 orc6 mcm2*
DNA conformation change	12	< 0.01	6.36	*hira mcm9 mcm5 mcm3 chd4b top2b nasp chaf1b mcm6 top1 mcm2 anxa1c*
Chromosome organization	25	< 0.01	4.27	*kdm5bb wrap73 hcfc1b hira mcm9 mcm5 mcm3 chd4b top2b rtel1 hmgn3 rad50 nasp l3mbtl2 chaf1b mcm6 hcfc2 prkdc top1 brd3b mcm2 uhrf1 anxa1c msh6 mre11a*
Cellular response to stress	27	< 0.01	2.65	*hsp90b1 rpa1 sel1l atf6 ercc4 pdia4 rad17 pold1 hsp70.1 rtel1 rad50 uggt1 hsp70l mcm6 sik1 eif2ak3 ercc8 prkdc spring1 nfe2l2b pold2 hmgb1a dusp1 mcm2 anxa1c msh6 mre11a*
Reg. of transcription, DNA-templated	59	< 0.01	1.89	*nfyal phc1 runx1t1 med17 rb1 zmynd8 rbl1 gata2b sox19a prkcbp1l hcfc1b atf6 hira ikzf1 znf710a taf5 cnot3b jdp2b safb foxd3 ssbp3b fosab zgc:65,895 cpsf1 hoxa13b hmgn3 ccar2 pdcd4b cebpb cux1b sox9b egr2a ahrrb l3mbtl2 ssbp4 si:dkey-27c15.3 snai1a hcfc2 znf740b rreb1a ftr80 bnc2 hoxc12a si:dkey-20i20.8 irf3 zgc:174,310 nkx3-1 supt20 mgaa si:ch211-160f23.5 deaf1 nfe2l2b sox4b hmgb1a jazf1a sirt6 uhrf1 e2f1 jak2a*
**Cell component**				
Mre11 complex	2	0.04	29.65	*rad50 mre11a*
Delta DNA polym. complex	2	0.04	29.65	*pold1 pold2*
MCM complex	4	< 0.01	25.41	*mcm5 mcm3 mcm6 mcm2*
MRNA cleavage factor complex	3	0.04	12.13	*wdr33 cpsf6 cpsf1*
DNA repair complex	3	0.05	11.12	*ercc4 ercc8 msh6*
Endoplasmic reticulum lumen	4	0.04	7.73	*pdia4 lrpap1 uggt1 p4ha1b*
Endoplasmic reticulum	28	< 0.01	2.77	*hsp90b1 sel1l atf6 eif2ak3 erap1a zdhhc16b tmed1b pdia4 tram1 sec23b serpinh1b tor1l3 tram2 lrpap1 ankle2 vti1b gnpnat1 piga porcnl smpd2b uggt1 elovl7a p4ha1b pigu calr zgc:114,200 calr3b ero1b*
Golgi apparatus	19	0.04	2.22	*cog5 zdhhc16b tmed1b lrpap1 golph3l vti1b gnpnat1 ift20 cux1b galnt7 arfrp1 slc35a2 arfip2a C3H17orf75 arfgef1 spring1 tmem241 arfgap3 b4galt1l*

**Figure 3:**
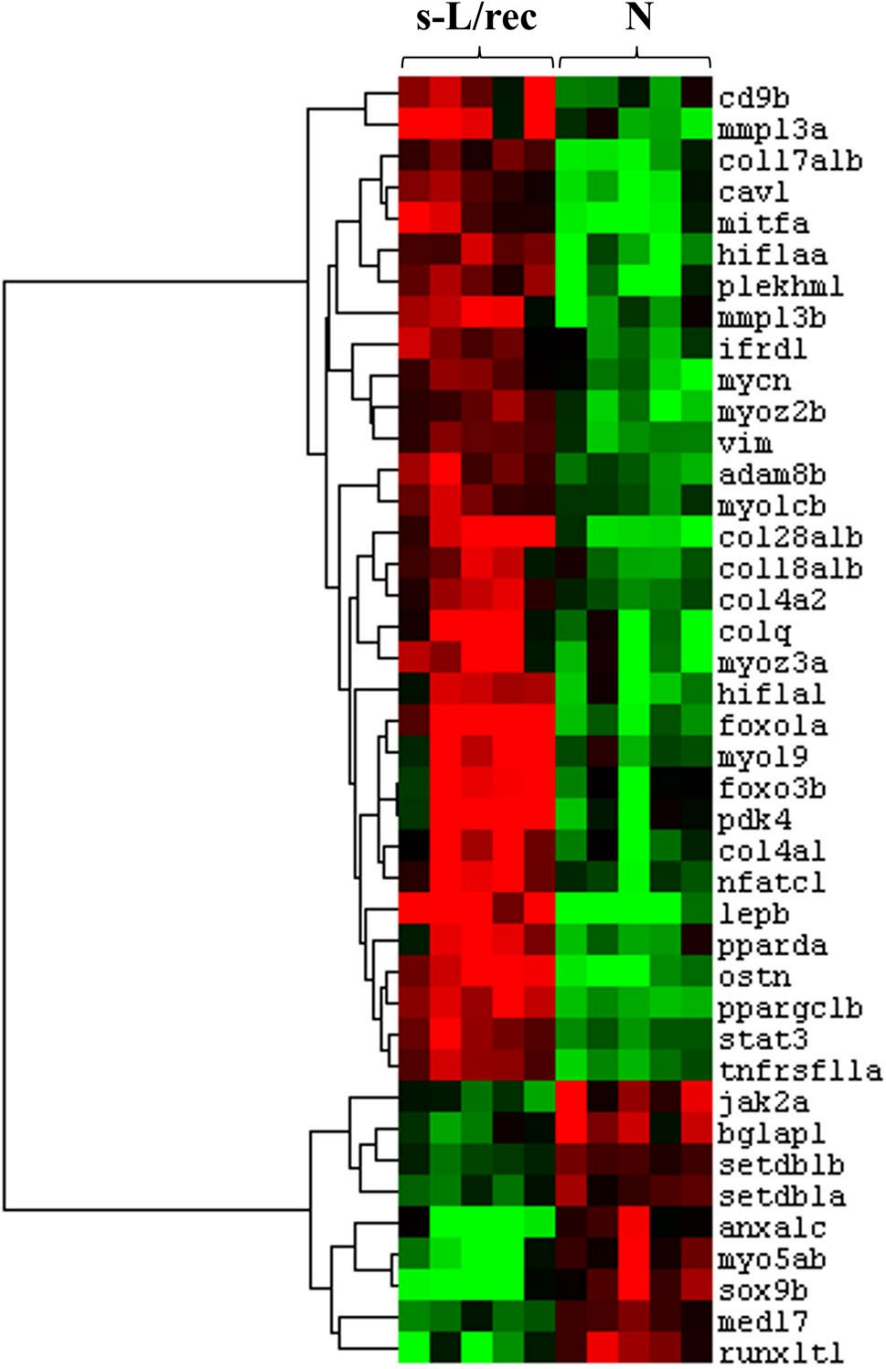
Heat map representing the relative mRNA expression levels of genes differentially expressed (*p* < 0.01) between normal (N) and recovered (s-L/rec) individuals, involved in bone remodeling and/or muscle regeneration.

Focusing on the 410 genes involved in bone remodeling or/and muscle regeneration, we found 41 genes exhibiting a differential expression between the s-L/rec and N-groups (*p* value < 0.01; Fig. 3, supplementary data 4). Out of the 41 differentially expressed genes, 32 were found up-expressed and 9 down-expressed in the s-L/rec group. Among the up-expressed genes *pdk4, ppargc1b, tnfrsf11a, mitfa, mmp-13, foxo1a,3b, ostn* all relied on osteoblast or osteoclast differentiation, were found among others. Others factors involved in ossification process such as *sox9b, setdb1 genes* and *bglapl* were down-expressed in the s-L/rec group.

Several factors involved in muscle regeneration were also found up-expressed genes in the recovered s-L/rec group. Among them *cd9b* involved in the fusion of myotubes^28^, whereas different factors implicated in muscular cell proliferation and/or differentiation (*ifrd1, stat3, cav1, pparda, foxo1a*). Some myogenic synthesis and maturation factors (*myoz2b*, *myoz3a, mycn, vim*) were also observed. In contrast, other genes involved in muscle development (*jak2a, myo5ab, anxa1c*) exhibited lower expression level in the s-L/rec group.

Several collagen genes encoding actors of the extra cellular matrix were also up-expressed in the s-L/rec group.

## Discussion

This is the first study documenting haemal lordosis recovery in zebrafish, in a time-dependent pattern. Recovery potential, internally and externally, can reach up to 74.5% within a week after the induction of a severe case of lordosis. Complete normal vertebrae shape and non-inverted spine orientation observed in this study, are in accordance with the existing literature reporting a partial to complete recovery of the vertebral column in lordotic gilthead seabream (27% of the recovered individuals^11^) and European sea bass (56% of the abnormal individuals^12^). No permanent injuries are expected to occur in the recovered animal’s vertebrae, since internal bone structures can be remodeled back to normal (present study). However it is still unclear whether the persisting damage of notochord tissues (present study) could have any adverse effect later in fish life. The absence of a counteracting kyphosis on our juveniles, previously highlighted to accompany lordosis recovery in other species^11^, can be possibly attributed to the different locomotion mechanisms behind swimming performance among the species.

Up to now knowledge attributes haemal lordosis induction on either failure of the axial skeleton to withstand the compressive loads induced by swimming^19^, or adaptation of the vertebral column to increased stress conditions due to increased muscle activities on the haemal region^21^. In both cases, increased exercise conditions are reported as the stimuli and an interaction between muscle and bone of the haemal region is described as the response center of changes leading to lordosis. Regardless of the species, a transfer to less intense swimming environment seems to trigger the recovery of this specific deformity (^11,12^, present study).

The transcriptome information obtained in this study provides a valuable resource for elucidating biological processes involved in the recovery of lordosis malformation in zebrafish. The RNA from which molecular analysis was performed was extracted from tissues with different cell types sampled in the haemal part of the fish. Therefore, we assume that the nature of the biological samples does not allow one to determine cell specific regulation of the regeneration/remodeling mechanisms taking place in the discrete part of tissue affected by lordosis. Nevertheless, our study shows that 973 genes (4% of all analyzed genes) exhibited differential RNA expression levels between the s-L/rec and N groups. Interestingly, we found within genes involved in chromosome organization and DNA metabolic process, several central players involved in the initiation of DNA replication such as *mcm* and *orc* genes. There is evidence that transcription of *mcm* (for minichromosome maintenance) genes increases during the G1 phase of cell cycle. It is also known that limited levels of MCM proteins may disrupt cell proliferation through the arrest of cell cycle progression at G1 phase and may be associated to the status of cell senescence^29,30^. A decrease of cell proliferation process in the haemal tissue sampled in the recovered group (s-L/rec) is supported by the down expression of *pcna* gene (*p* = 0.019) (supplementary data 1). The over-representation of actors related to chromosome organization and to the regulation of transcription that we observed within genes down-expressed in the recovered group is consistent with the fact that senescence associated-chromatin structural changes affect genome accessibility and their transcriptional program^31^. Cellular senescence is recognized as a fundamentally important biological process when transiently induced in the dynamic of tissue regeneration in different vertebrate species including zebrafish^32–34^. One reason explaining the beneficial effect of senescent status is that cells produce a variety of senescence-associated secretory phenotype (SASP) markers including cytokines, chemokines, growth, extracellular matrix components (ECM) and matrix metalloproteinases which can contribute to tissue repair^35,36^. Indeed the present transcriptomic data reveal a significant regulation of processes related to SASP such as chromosomal organization and up-expression of various genes related to the synthesis of extracellular matrix components, including collagens (*col4a2, col4a1, col18a1b, col28a1b, col17a1b*), fibronectin (*fn1b, itga5*), proteoglycans (*galnt16*), glycosaminoglycans (*ndst1a*) and chemokines (cxcl2a, cxcl18b) in the s-L/rec group. On the contrary, DNA repair complex process was found enriched within genes downregulated in this group. Therefore, additional complementary analysis based on beta-galactosidase and/or lipofuscin staining^37^ and quantification of protein markers involved in cell cycle regulation (e.g. P53 and P21) are necessary to confirm whether the cellular senescence process is involved in the present recovery process of haemal lordosis.

The *per* genes that we found upregulated in the haemal part of the s-L/rec fish are central elements of a transcriptional feedback loop that generates circadian rhythms. Circadian machinery is known to confer rhythmicity to a large part (10%) of the transcriptome^38^, including genes involved in cell senescence^39^. The over-representation of factors involved in circadian regulation that we observed, together with the regulation of chromosomal organization process, agrees with previous studies indicating interrelated and intricated regulations between chromatin remodeling and the circadian clock process^40,41^.

Interestingly, we found among the regulated transcription factors some key genes intricately involved in adipogenesis, muscular regeneration and bone remodeling. The regulation of *ppargc1b, pparda* genes known to be involved in adipogenesis^42^ may be related to the abundant quantity of adipose tissue that we observed inside the bone marrow spaces of haemal part in recovered fish. Indeed, when arising, bone marrow spaces are known to be filled with adipose tissue^14^. Recent discoveries of the secretory and metabolically active profile of bone marrow adipose tissue^43^, suggest that its regulation could promote skeletal regeneration^44^. We also observed, in lordotic recovered group, up expression of adiponectin (*adipoq*), its receptor (*adipor2*) and leptin (*lepb*), adipocytokines reported to be expressed in adipose tissue of zebrafish^45^, known to interfere energy metabolism^44^.

In zebrafish, as in mammals, adipogenesis and osteogenesis depend on intricated molecular regulations driven by transcription factors PPARG and RUNX2 which are involved in the differentiation of mesenchymal stem cells (MSC) into adipocytes and osteoblasts, respectively^46^. The fact that *runx2* gene was not differentially expressed in the present study (*p* = 0.97 and *p* = 0.37 for *runx2a* and *runx2b*, respectively) suggested that the regulation of this transcription factor was not involved within the haemal lordotic recovery process. Nevertheless, we found differentially expressed several genes related to bone cells differentiation (*pdk4, ppargc1b, tnfrsf11a, mitfa, mmp-13, foxo1a,3b, ostn, nfatc1a*). Of particular interest is the regulation of the microphthalmia-associated transcription factor (*mitf*) which is especially involved in bone remodeling and more specifically in vertebrates osteoclastogenesis^47,48^. The up-expression of *mitf* gene, together with some of its known target genes such as *tnfrsf11a* which encodes Rank-l receptor in osteoclast and *nfatc1a* involved in osteoclast differentiation that we observed in the present study, suggest that osteoclastogenesis process is stimulated during the recovery of haemal lordosis^49^. Interestingly, the expression of *mitf* gene, that is transcriptionnaly regulated by clock genes, has been shown involved in circadian physiology in zebrafish^50,51^. These data suggest that bone remodeling and clock machinery processes may be intricately regulated during the present recovery process. According to that, the circadian clock regulates bone turnover in mammals and a role for the circadian clock in the timing of tissue regeneration has been already demonstrated in zebrafish^52–54^. Involvement of bone remodeling-related process in the haemal lordosis recovery was also revealed in the present study by the regulation of genes encoding protein of the bone extracellular matrix proteins (*osn, collagens)* and genes involved in mineralization (*bglap)*. Collagen fiber orientation differences between our groups, observed by means of histology, could potentially be linked to this regulation.

The up-expression of the *pparda* expression in recovered fish though, may also concern regulation occurring in muscular tissue. In response to increased swimming exercise, vertebral body end plates, of both exercised-normal and exercised-induced-lordotic vertebrae, have previously been reported to experience the highest muscular stress concentration^1,18^. *Pparda* gene was shown to be involved in exercise-induced fast skeletal muscle fiber remodeling in zebrafish^55^. Rovira and collaborators^55^ revealed that muscle metabolism and remodeling was related to the regulation of the *ppargc1a* gene expression as well as of some of its targets, such as *pparda* and *myosin chains.* Interestingly, we also found *ppargc1a* gene up-expressed in the recovered fish (*p* = 0.02) and myosin chain genes up-expressed (*smyhc2, myl2a, myl12.2*) or down-expressed (*myh9a*) suggesting a fiber-type switch toward the remodeling process. In mammals, Angione and collaborators^56^ showed that *ppard* acts, through another transcription factor Foxo1, in regulating muscle progenitor cells and postnatal muscle regeneration^56^. We also found *foxo1* gene up-expressed in the recovered fish. We assume that the regulation of the molecular pathways involving transcriptional factors *pparda*, *ppargc1a* and *foxoa1* supports the regulation of the different target genes implicated in muscular cell proliferation and/or differentiation (*ifrd1, stat3, cav1*) and some myogenic synthesis and maturation factors (*myoz2b*, *myoz3a, mycn, vim*), contributing to haemal lordotic recovery.

It is admitted in mammalian and non-mammalian species, including fish, that tissue repair is a tightly coordinated comprisal of multiple cell types and overlapping processes that broadly include inflammation, cell proliferation, differentiation and migration, and remodeling^57,58^. It involves the dynamic interactions of cells from various lineages (e.g. muscle, bone, fibroblast) with components of the extracellular matrix (ECM) and growth factors^59–61^. The present study is the first evidence indicating a potential tissue repair process behind the recovery of lordosis in zebrafish juveniles. Several key genes of cellular senescence, circadian clock and adipogenesis regulation seem to be differentially expressed behind this musculoskeletal recovery procedure. Simultaneously several genes already reported to be involved in bone or muscle remodeling are highlighted. Future tissue-specific studies are necessary to clarify the exact molecular mechanisms implied in lordosis recovery. In addition, a transcriptomic comparison between s-L, s-L/rec and N individuals could be interesting to complete the knowledge on the induction mechanism of lordosis. Overall, zebrafish potential as a model organism to study the fast bone and muscle changes behind a skeletal deformity imposed by mechanical stress is verified. And since zebrafish shares similar skeletal cells and ossification types with mammals, an emerging role in regenerative medicine of the musculoskeletal system arises^62^.

## Materials and methods

**Figure 4:**
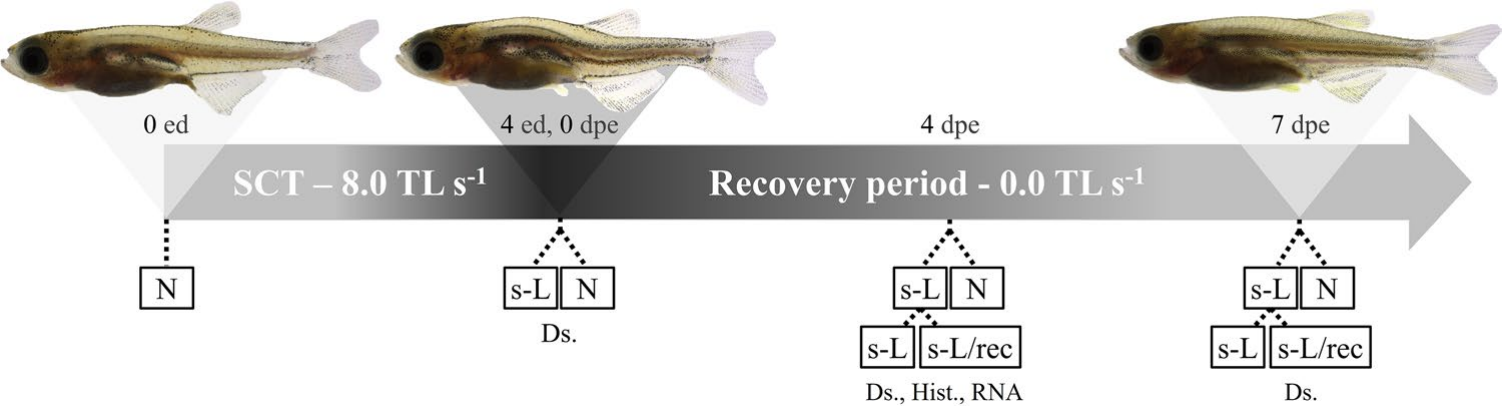
Basic experimental design. Juvenile zebrafish with normal external morphology were subjected to a swimming challenge test (SCT) for the induction of haemal lordosis. The severe lordotic (s-L) and normal (N) fish were monitored for a week under almost 0.0 TL s^−1^. Lordosis recovery (s-L/rec) was examined by means of double staining for bone and cartilage (Al.), histology (Hist.) and gene expression analysis (RNA). ed, exercise days. dpe, days post exercise.

The experimental design depicted in Fig. 4 enabled the examination of haemal lordosis recovery potential. For lordosis induction, juvenile zebrafish (12.2 ± 0.6 mm TL, total length) were exposed to a 4-day swimming challenge test (SCT) at 8.0 TL s^−1^ swimming speed (corresponding to 56% of the species' critical swimming speed^63^). Exposed fish were then allowed to recover for a period of one week under almost 0.0 TL s^−1^. Monitoring of the changes on the lordotic vertebral column was performed individually by means of whole mount staining, histology and gene expression analysis. All trials were performed in three independent replicates derived from a common batch of eggs.

### Animal origin and experimental setup

Approximately one thousand fertilized embryos were collected from a common brood stock of 250 wild-type zebrafish (ZF WT; Wageningen Agricultural University, Wageningen, the Netherlands), randomly divided in three replicates and placed inside three cubic net pens (4.5 L volume each). Net pens were located in a common aquarium of 40 L volume equipped with a closed recirculation system. A feeding regime including *Artemia* nauplii (5–11 dph, days post hatching) and a commercial dry microdiet (> 8 dph, Zebrafeed, Sparos Lda, Olhao, Portugal) was applied (supplementary table S1).

Juveniles reaching 12.0–12.5 mm TL (25–30 dpf) marked the beginning of the SCT. Thirty fish per replicate were selected to constitute the experimental groups. Before SCT, fish were anaesthetized (2-phenoxyethanol, C = 0.2–0.4 mL L^−1^), measured for TL (Lumenera Infinity Analyze Microscopy Software, version 6.5.4, Ottawa, Canada), photographed under a stereoscope (Olympus SZ16) and examined for the presence of any axial abnormalities. Only fish without any gross abnormalities were used. SCT was performed according to Printzi et al.^1^ in three swimming channels (one channel per replicate). Shortly, fish were subjected to a 4-day continuous exercise regime at 8.0 TL s^−1^, with short cut offs (15 min) during feeding periods. Swimming tunnels (70 cm length, 10 cm depth, 5 cm width) were connected to closed recirculation systems, consisting of two holding tanks (40–100 L volume each). Water velocity was measured and adjusted daily at 8.0 TL s^−1^.

At the end of SCT, the procedure previously described was used to anaesthetize, photograph and examine all the exercised individuals for the presence of haemal lordosis. Fish categorization was based on the presence of the characteristic dorsal shift of the caudal peduncle in severe lordosis (s-L group, supplementary Fig. 1a). Fish lacking this shift were considered as normal (N, supplementary Fig. 1c), whereas any individual without the typical lordotic or normal external shape^1^ was classified as light lordotic (l-L, supplementary Fig. 1b). Once categorized, fish from each replicate were pooled together to constitute a total of 43 severe lordotic (s-L, 49% of the exercised fish), 31 light lordotic (l-L) and 13 normal fish (N). Three individuals were accidentally lost, due to handling error during the swimming test. Light lordotic individuals (l-L) were directly euthanized (overdose of 2-phenoxyethanol, 0.5 mL L^−1^), fixed in formalin and stained for bone and cartilage^64^ to verify their external categorization, before being excluded from the following experimental tasks.

Throughout the rearing and SCT period, the closed recirculation systems maintained the abiotic parameters at 28.0 °C (± 0.5 °C), 500–700 μS cm^−1^ conductivity, 7.0–7.5 pH and > 90% oxygen saturation. The photoperiod was set at 14/10 h light to dark.

### Lordosis monitoring during the recovery period

Studied phenotypic groups (s-L and N) were placed in 3 L (2 s-L fish per tank separated with a divider) or 8 L (5–8 N fish per tank) standard zebrafish aquariums (Zebtec Standalone, Techniplast, Italy) to recover from swimming exercise. The recovery of lordotic external phenotype was examined at 4 and 7 days post-exercise (dpe), following the anesthetization (2-phenoxyethanol, C = 0.2–0.4 mL L^−1^) and photography of all the juveniles under a stereoscope (Olympus SZ16). The same procedure was followed for monitoring the N group. At four dpe, a random sample of fish with normal (N) and with a recovered phenotype (s-L/rec) were taken for histological examination and RNA analysis. The rest of the individuals returned to their tanks for another three recovery days. During the recovery period, feeding was performed three times per day. Abiotic conditions were maintained at the same levels with the larval rearing period.

To validate the external categorization at each critical time point 3–7 fish per phenotypic group (N, s-L, s-L/rec, at 0 and 4dpe) and all the remaining fish at 7 dpe were euthanized (overdose of 2-phenoxyethanol, 0.5 mL L^−1^), photographed, fixed in formalin and stained for bone and cartilage^64^.

Recovery frequency between 4 and 7 dpe was tested by means of G-test^65^.

### Histology analysis

At four dpe, haemal parts of three juveniles with a recovered external lordotic phenotype from the severe group (s-L/rec) and three normal fish (N) were dissected. After tissue embedding in glycol methacrylate (GMA), parasagittal sections of 2–5 μm were obtained in the center of the previously abnormal area (S) and respective area on normal (N). Sections were stained with toluidine blue (0.5% toluidine blue), mounted on glass slides and cover slipped to allow the follow up observation under a Carl Zeiss Axio Imager Z microscope.

### RNA extraction and sequencing

At four dpe, six individuals from each group (s-L/rec, N, after the pool of the replicates), with normal external phenotypes were randomly sampled. The dissected haemal parts of the juveniles were preserved in RNA Stabilization Reagent (RNAlater, Qiagen, Hilden, Germany). The dissected haemal samples included the tissue located between the cross-section at the level of the anterior anal-fin ray and the cross-section at the base of caudal-fin rays (supplementary Fig. 2). Total RNA extraction was achieved by Extract-all reagent (Eurobio; Courtaboeuf, Essonne, France) combined with Nucleospin RNA column which includes one step of DNase treatment (Macherey–Nagel, Düren, Germany). Extracted RNA concentration and purity were verified using an ND-1000 NanoDrop® spectrophotometer (Thermo Scientific Inc., Waltham, MA, USA). An Agilent Bioanalyzer 2100 (Agilent Technologies Inc., Santa Clara, CA, USA) allowed the visualization of an RNA integrity (RIN) score greater than nine in all samples.

From the extracted RNA samples, five from each group were selected based on the quality of the RNA and they were sent for transcriptome analysis via RNA sequencing at GenomiX (MGX, Montpellier, France) following the procedure described by Cohen-Rengifo et al.^66^ Total RNA was quantified on Fragment Analyzer (Agilent Technologies, Santa Clara, CA, USA) using the standard sensitivity RNA kit (DNF-471-0500). Libraries were constructed using Stranded mRNA Prep Ligation kit (Illumina, San Diego, CA,USA) according to the manufacturer’s instructions. Briefly, poly-A RNAs were purified using oligo(dT) magnetic beads from 530 ng of total RNA. Poly-A RNAs were fragmented and underwent a reverse transcription using random hexamers. During the second strand generation step, dUTP substitued dTTP in order to prevent the second strand to be used as a matrix during the final PCR amplification. Double stranded cDNAs were adenylated at their 3' ends and ligated to Illumina's universal anchors. Ligated cDNAs were amplified following 12 PCR cycles. During this PCR, the libraries were indexed and the adapter sequences were completed in order to be compatible with the cluster generation step. PCR products were purified using AMPure XP Beads (Beckman Coulter Genomics, Brea, CA, USA). Libraries were validated using Standard Sensitivity NGS kit (DNF-473-0500) on Fragment Analyzer and quantified using KAPA Library quantification kit (Roche, Bâle, CHE).

RNA raw data are available in EBI public database (PRJEB56543). Sequences were aligned to the Danio rerio genome (GRCz11) using the splice junction mapper, TopHat2 2.1.1^67^ (with Bowtie 2.3.5.1^68^) on a set of gene model annotations (gff file downloaded from NCBI on 25/03/2020). Final read alignments having more than 6 mismatches were discarded. Samtools (v1.9) was used to sort the alignment files. Then, gene counting was performed with Featurecounts v.2.0.0)^69^. As the data are from a strand-specific assay, the reads have to be mapped to the opposite strand of the gene (-s 2 option). Before statistical analysis, genes with less than 25 reads (cumulating all the analysed samples) were filtered out. Differentially expressed genes were identified using the Bioconductor^70^ package DESeq2 v1.26.0^71^ (R version 3.6.1). Data were normalized using the DESeq2 normalization method. An adjusted *p* value was calculated according to the FDR method from Benjamini–Hochberg for each gene. Genes data with a *p* value cut-off at 0.01 to limit false positive rate were afterwards imported at ShinyGo v0.76 software^72^ to gain enriched gene ontologies (GO) behind significantly up or down expressed genes between (s-L) and (N) individuals. False discovery rate (FDR) was calculated based on nominal P-value from the hypergeometric test for the graphs and set at 0.05. A focus was put on the genes involved in bone remodeling and muscle regeneration based on functional annotations available on Amigo website (http://amigo.geneontology.org/amigo) completed with bibliographic research.

### Ethical statement

All the experimental procedures involving zebrafish were performed in accordance with Greek (PD 56/2013) and EU (Directive 63/2010) legislation for animal experimentation and welfare. The Animal Care Committee of the Biology Department of the University of Crete approved all protocols (Permit number: 125661/22). All methods are reported in accordance with ARRIVE guidelines.

## Supplementary Information

Supplementary Information 1.

Supplementary Information 2.

Supplementary Information 3.

Supplementary Information 4.

Supplementary Information 5.

Supplementary Information 6.

Supplementary Information 7.

## Data Availability

All data generated or analyzed during this study are included in this published article and in the public database https://doi.org/10.12770/4691cb43-8da5-4542-ac8f-2beb6aeb2929 (and its Supplementary Information files).
