# Supplementary material for: Juvenile zebrafish (Danio rerio) are able to recover from lordosis
Source: Sci Rep. 2022 Dec 13;12:21533. doi: 10.1038/s41598-022-26112-2 (PMC9748118; doi:10.1038/s41598-022-26112-2)
Supplement: Supplementary file 5 — Supplementary Information 5. [file 41598_2022_26112_MOESM5_ESM.docx]

Lordosis recovery in juvenile zebrafish (*Danio rerio*)

A. Printzi^1,2^ *, D. Mazurais^2^, P.E. Witten^3^, L. Madec^2^, A-A. Gonzalez^4^, X. Mialhe^4^, J-L. Zambonino-Infante^2^, G. Koumoundouros^1^

^1^ Biology Department, University of Crete, Crete, Greece.

^2^ IFREMER, University of Brest, CNRS, IRD, LEMAR, F-29280, Plouzané, France

^3^ Department of Biology, Gent University, Gent, Belgium

^4^ MGX-Montpellier GenomiX, Univ. Montpellier, CNRS, INSERM, Montpellier France

*, to whom correspondence should be addressed

Table S1. Feeding regime per net pen during the experimental trial. Artemia nau, Artemia nauplii. df, dry food. dpf, days post fertilization. dpe, days post exercise.


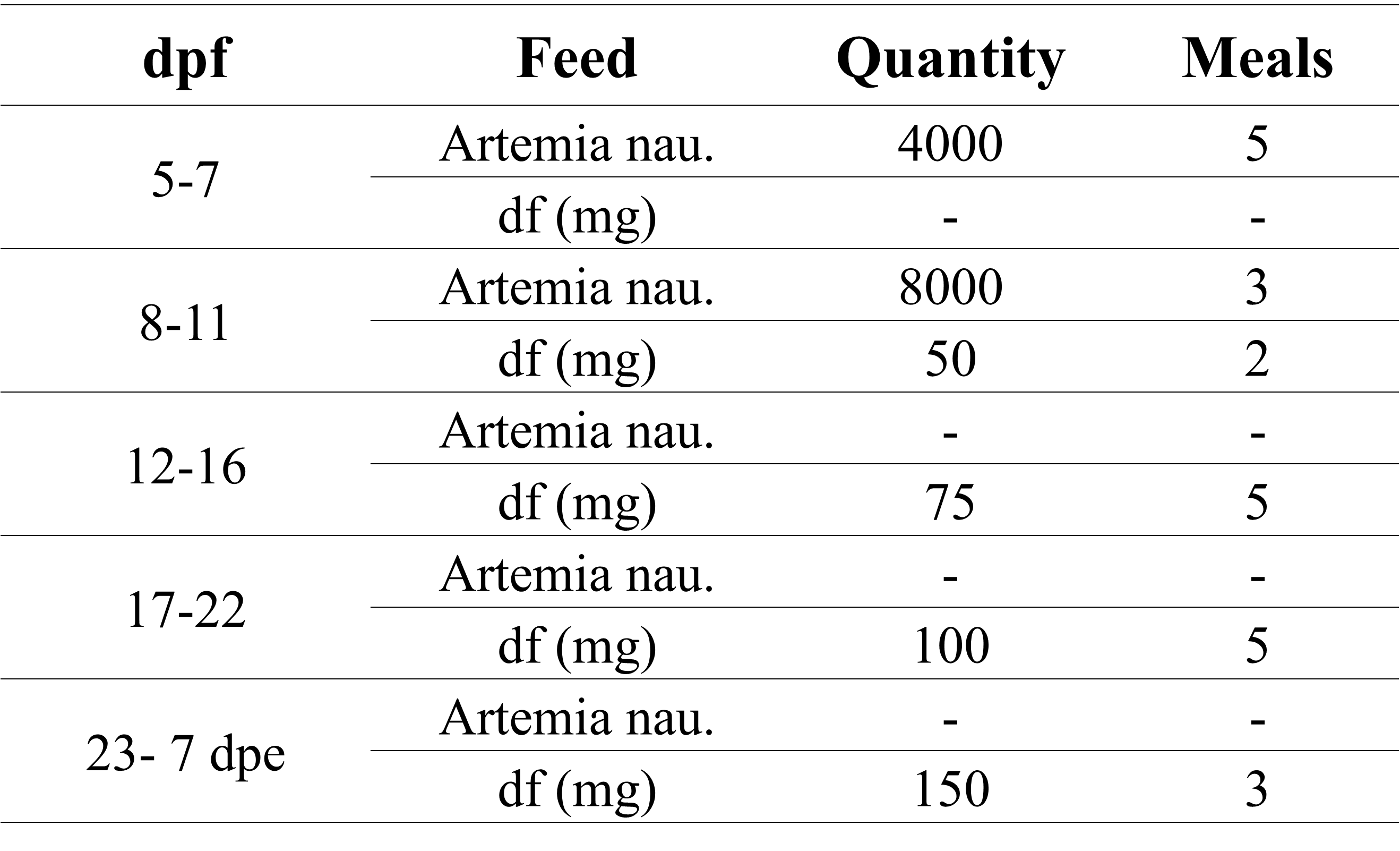


Figure S1. Representative cases of the external phenotype categorization on severe lordotic (s-L, a), light lortotic (l-L, b) and normal (N, c). Scale bars equal to 1mm.

Figure S2. Representation of the dissected haemal parts sampled for histology and RNA analyses. The exact sample location falls within the spotted line. Scale bar equals to 1mm.
