# Supplementary figures and images for: Juvenile zebrafish (Danio rerio) are able to recover from lordosis
Source: Sci Rep. 2022 Dec 13;12:21533. doi: 10.1038/s41598-022-26112-2 (PMC9748118; doi:10.1038/s41598-022-26112-2)

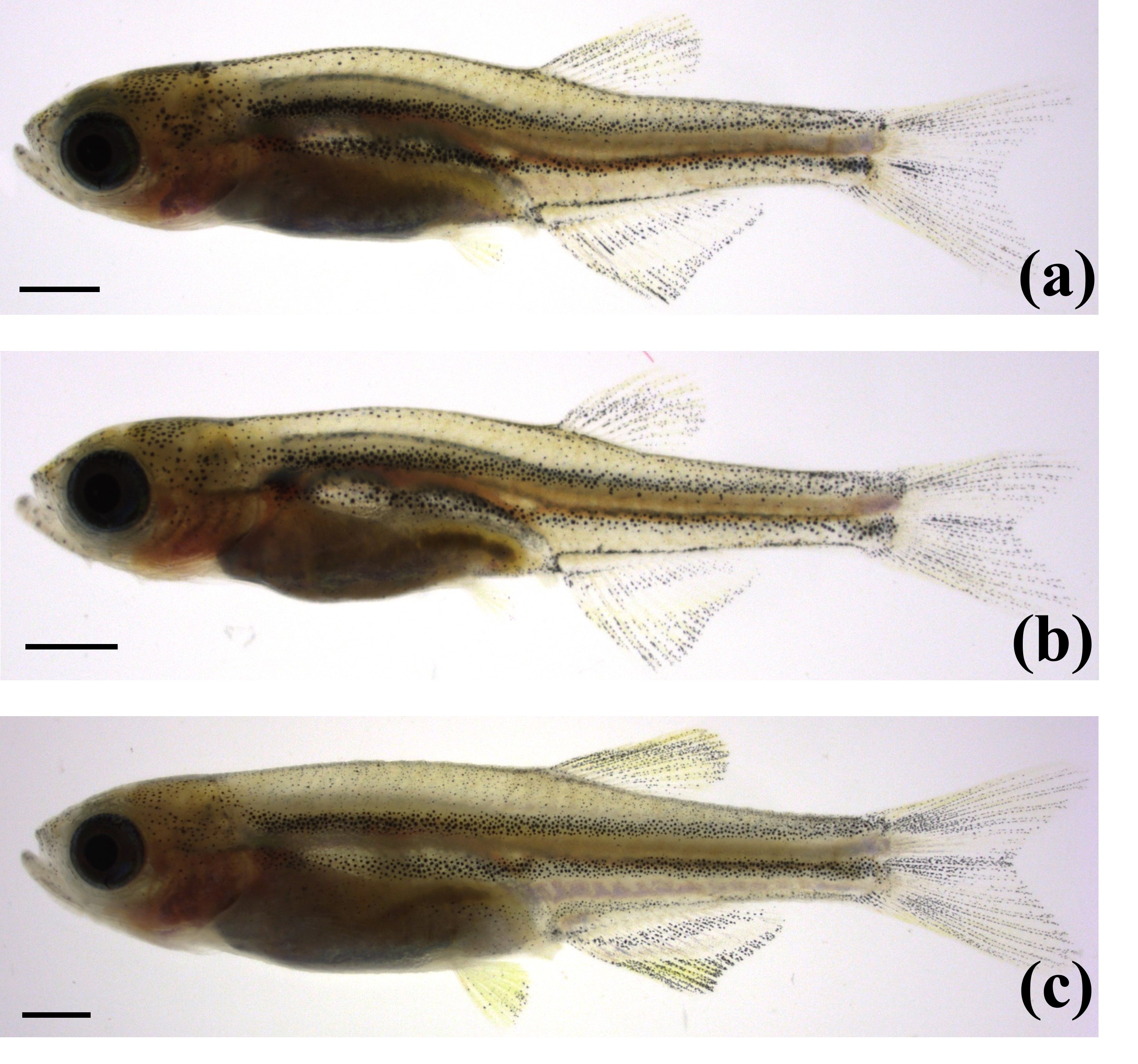

Supplement: Supplementary file 6 — Supplementary Information 6. [file 41598_2022_26112_MOESM6_ESM.jpg]

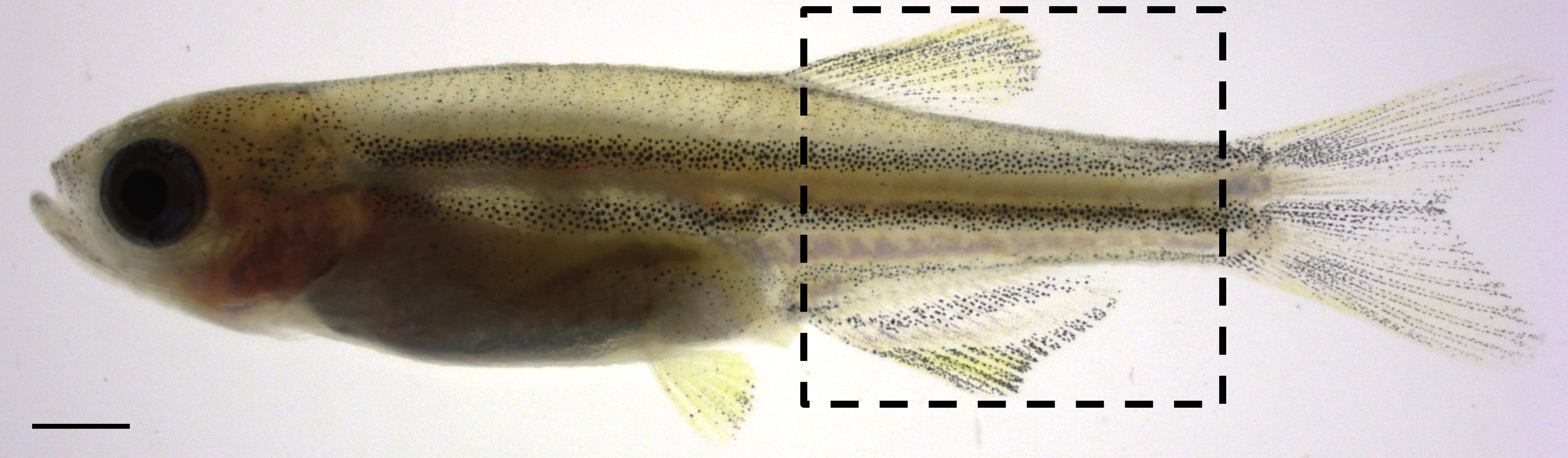

Supplement: Supplementary file 7 — Supplementary Information 7. [file 41598_2022_26112_MOESM7_ESM.jpg]
